# A novel highly selective allosteric inhibitor of tyrosine kinase 2 (TYK2) can block inflammation- and autoimmune-related pathways

**DOI:** 10.1186/s12964-023-01299-7

**Published:** 2023-10-16

**Authors:** Celia X.-J. Chen, Wei Zhang, Shulan Qu, Fucan Xia, Yidong Zhu, Bo Chen

**Affiliations:** 1 Department of Immunology and Inflammation, Shanghai Qilu Pharmaceutical R&D Center Limited, Shanghai, China; 2 Present address: China Resources Pharmaceutical Group Limited, Beijing, China

**Keywords:** TYK2, JAK, Cytokine pathway, Pseudokinase regulatory domain, Allosteric inhibitor, Psoriasis, Autoimmune diseases

## Abstract

**Background:**

As a member of the Janus kinase (JAK) family, which includes JAK1, JAK2 and JAK3, tyrosine kinase 2 (TYK2) plays an important role in signal transduction and immune system regulation. Moreover, it is also involved in the development of many types of inflammatory and autoimmune diseases, such as psoriasis and systemic lupus erythematosus (SLE). TYK2 is an attractive therapeutic target, and selective inhibition of TYK2 over other JAK family members is critical for the development of TYK2 small molecule inhibitors. However, targeting the catalytic region of the TYK2 ATP-binding site is a major challenge due to the high structural homology between the catalytic regions of the JAK family proteins.

**Results:**

In this study, we developed a novel small molecule inhibitor (QL-1200186) by targeting the pseudokinase regulatory domain (Janus homology 2, JH2) of the TYK2 protein. The binding sites of QL-1200186 were predicted and screened by molecular docking. The inhibitory effects on IFNα, IL-12 and IL-23 signaling were tested in cell lines, human peripheral blood cells and human whole blood. The pharmacokinetic (PK) and pharmacodynamic properties of QL-1200186 were verified in mice. QL-1200186 showed high affinity for TYK2 JH2 and had no apparent selectivity for the TYK2 and JAK homologous kinase domains; these effects were demonstrated using biochemical binding, signaling pathway transduction (JAK1/2/3) and off-target effect assays. More importantly, we revealed that QL-1200186 was functionally comparable and selectivity superior to two clinical-stage TYK2 inhibitors (BMS-986165 and NDI-034858) in vitro. In the PK studies, QL-1200186 exhibited excellent exposure, high bioavailability and low clearance rates in mice. Oral administration of QL-1200186 dose-dependently inhibited interferon-γ (IFNγ) production after interleukin-12 (IL-12) challenge and significantly ameliorated skin lesions in psoriatic mice.

**Conclusion:**

These findings suggest that QL-1200186 is a highly selective and potent inhibitor of TYK2. QL-1200186 could be an appealing clinical drug candidate for the treatment of psoriasis and other autoimmune diseases.

Video Abstract

**Supplementary Information:**

The online version contains supplementary material available at 10.1186/s12964-023-01299-7.

## Background

Janus kinases (JAKs) are multidomain tyrosine kinases that mediate cytokine transduction and regulate the immune system. The JAK family consists of four members, including JAK1, JAK2, JAK3 and tyrosine kinase 2 (TYK2), which are involved in different cytokine signaling pathways [1, 2]. When cytokines bind to their receptors to activate the JAK signaling pathway, signal transducer and activator of transcription (STAT), as the substrate of the JAK family, is phosphorylated by JAKs to form dimers, which then pass through the nuclear envelope into the nucleus for transcriptional regulation. This pathway is known as the JAK-STAT signaling pathway, which plays an important role in the regulation of the immune system [3]. JAK transduction is mediated by a range of interleukin (IL) receptors, interferon (IFN) receptors, colony-stimulating factors (CSFs) and hormones. For instance, IL-2, IL-4, IL-7, IL-9, IL-15 and IL-21 can activate the JAK1/3 signaling pathway through their receptors, whereas IL-3, IL-5, granulocyte–macrophage colony-stimulating factor (GM-CSF) and thrombopoietin can activate the JAK2 signaling pathway, all of which have been shown to be pathogenic pathways for different autoimmune diseases [4].

In recent years, several small molecule drugs targeting JAKs have been approved and provide great benefits to many patients with inflammatory disorders. However, the chronic use of current nonselective JAK inhibitors is limited due to safety concerns. Indeed, inhibition of JAK1-3 has been associated with an increased risk of serious infections, cardiovascular problems and cancer. Due to these serious side effects, the FDA requires a black box warning on these drugs [5, 6]. Given this situation, the unmet clinical need remains high for patients with autoimmune diseases.

As a member of the JAK family of nonreceptor tyrosine kinases, TYK2 can be activated by IL12, IL-23 and type-I IFNs. This action is believed to be an important cellular mechanism for the development of autoimmune diseases [7, 8]. For example, TYK2-deficient mice have been reported to be viable but sensitive to viral infections. Additionally, TYK2-mediated signaling can increase the number and enhance the function of T helper type-1 (Th1) and Th17 cells, suggesting that TYK2 may be involved in innate and acquired immune responses [9–11]. Moreover, mice harboring TYK2 polymorphisms show different susceptibilities to collagen-induced arthritis, thereby demonstrating that TYK2 deficiency leads to clinical rheumatoid arthritis [12, 13]. In addition, TYK2 deficiency has been shown to reduce disease scores and lymphocyte infiltration in the inflamed central nervous system in an experimental model of autoimmune encephalomyelitis [14]. These results indicated that inactivation of the *TYK2* mutation could provide protection against various autoimmune diseases, including psoriasis and inflammatory bowel disease (IBD) [15–17]. Therefore, targeted intervention of TYK2 via small molecule inhibitors has been shown to be a viable option for the treatment of autoimmune diseases.

**Fig. 1:**
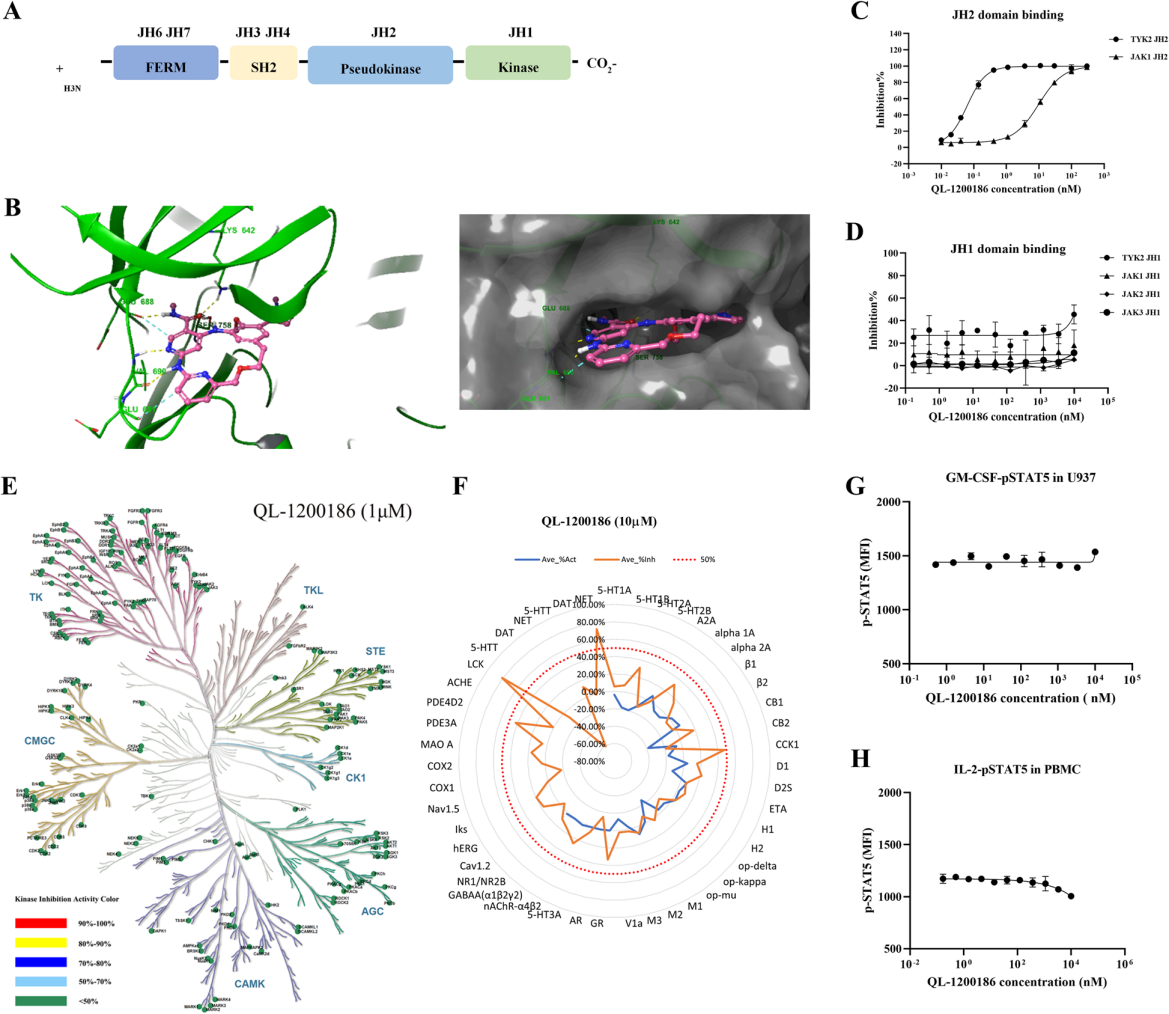
QL-1200186 can bind TYK2 JH2 with high selectivity and has suitable safety in vitro. **A** Structural illustration of the TYK2 domains. **B** Docking model of QL1200186 with TYK2 JH2. TYK2 JH2 ribbon and carbons are shown in green and QL-1200186 carbons are shown in pink. TYK2 JH2 surface is shown in transparent gray. **C** The binding of QL-1200186 to JH2 of TYK2 and JAK1. **D** The effects of QL-1200186 on JAK1/2/3 and TYK2 JH1 kinase activities determined by HTRF. **E** A kinase selectivity panel was screened by the ADP-Glo™ kinase assay. **F** The pharmacological safety of QL-1200186 in vitro was determined using a 44 safety target panel. **G** GM-CSF-induced pSTAT5 production in U937 cells detected by FACS. **H** IL-2-induced pSTAT5 levels in human PBMCs detected by FACS

TYK2 is a complex protein with multiple domains (Fig. 1A). Unlike other kinase domains, the pseudokinase domain of TYK2 (JH2) lacks catalytic activity. However, JH2 plays a key role in the receptor-mediated activation of adjacent catalytic domains (JH1) through autoinhibitory interactions. It has been reported that stabilizing the TYK2 JH2 domain by small molecules can cause a protein conformational change that inhibits JH1 domain-mediated catalytic activity [18]. This stabilization leads to the inhibition of the TYK2-mediated signal transduction cascade that includes the IL-12, IL-23 and type-I IFN pathways [19]. Previous studies have shown that the P1104A coding variant in the TYK2 gene has protective effects against a variety of autoimmune diseases [20–22]. TYK2-selective inhibitors developed by targeting the TYK2 JH2 domain, such as BMS-986165 (deucravacitinib) and NDI-034858, have shown suitable safety profiles in clinical trials [23, 24].

In this study, we developed a highly selective and potent small molecule inhibitor of TYK2 (QL-1200186) to treat autoimmune diseases. Its profile suggested that further clinical development could be possible.

## Materials and methods

### Molecular docking

The structures of TYK2 JH2 (Protein Database ID: 6NZP) and QL-1200186 were prepared using Protein Preparation Wizard and LigPrep modules, respectively. Then, the binding site was defined as a 20 × 20 × 20 Å3 cubic box centered on the ligand of the TYK2 JH2 protein. Afterward, molecular docking was undertaken using Glide standard precision, and flexible macrocycle sampling was adopted. Postdocking minimization was undertaken to further refine the docking results. All of the modules mentioned were tools included in Schrödinger 2021–3 software (https://www.schrodinger.com/).

### Assay to measure TYK2 pseudokinase domain binding

An assay that measures the ability of a compound to bind to the TYK2 pseudokinase domain through competition with a tracer was employed. First, human polyhistidine-tag (his-tagged) TYK2 pseudokinase was added to different concentrations of QL-1200186. Then, his-terbium-labeled antibody in assay buffer (HEPES (20 mM), pH 7.5, MgCl_2_ (10 mM), 0.015% Brij-35, dithiothreitol (2 mM) and bovine serum albumin (50 μg/mL)) was added to the recombinant TYK2 pseudokinase domain. Next, fluorescently labeled BMS-986165 analog tracers were added, followed by centrifugation for 30 s and incubation for 60 min at room temperature (RT). After 1 h at RT, the homogeneous time-resolved fluorescence (HTRF) signal was measured on a plate reader (Envision™; PerkinElmer, Waltham, MA, USA).

### Assay to measure JAK1/2/3 and TYK2 activity

We measured the ability of a compound to bind to JAK1/2/3 and the TYK2 JH1 domain by using HTRF® KinEASE™-TK kits (Millipore, Bedford, MA, USA). JAK1/2/3 and the TYK2 JH1 domain in assay buffer were added to various concentrations of QL-1200186 and incubated for 15 min at RT. Then, the plate was incubated with the biotin-labeled TK substrate and adenosine triphosphate for 45 min at RT. Next, SA-XL665 and TK antibody-Eu3 were added to the detection buffer and incubated for 1 h at RT. The HTRF signal was measured on a plate reader (Envision).

### Kinase panel assay

The selectivity of QL-1200186 was measured using a panel of 207 kinase proteins. QL-1200186 (final concentration = 1 μM) was placed in a 384-well plate. Different kinase solutions were configured in kinase buffer, HEPES (50 mM), MgCl_2_ (10 mM), 0.01% Brij, dithiothreitol (2 mM), and then transferred to 384-well plates. After incubation for 10 min at RT, the substrate and adenosine triphosphate were added, and the reaction was allowed to continue for 60 min. Kinase selectivity was determined by the ADP-Glo™ Kinase Assay (V9103; Promega, Fitchburg, WI, USA). Finally, a microplate reader (BMG Labtech, Ortenberg, Germany) was used to measure the luminescence signal. After data analysis, heatmaps and kinase selection trees were drawn using Prism 8.0.2 (GraphPad, La Jolla, CA, USA) and KinMap (www.kinmap.org/), respectively.

### Assay to measure off-target effects

The pharmacologic safety of QL-1200186 was tested by analyzing forty-four safety targets in vitro. The off-target effects of QL-1200186 on 24 G protein-coupled receptors (β1, β2, D1, H2, A2A, CB1, CB2,5-HT1A, 5-HT1B and D2S) were evaluated by measuring the changes in cyclic adenosine monophosphate and IP-1 levels. The effects of QL-1200186 on the current changes of eight ion-channel targets were determined by methods based on fluorescent imaging plate reading and patch clamps. Using stable transfer or transient plasmid transfer, the excitatory and inhibitory effects of QL-1200186 on glucocorticoid receptors and androgen receptors in the nucleus were determined by the change in luciferase expression. The inhibitory effects of QL-1200186 on seven enzymes (including cyclo-oxygenase-2, phosphodiesterase (PDE) 3A and PDE4D2) were evaluated by biochemical methods based on luminescence and fluorescence. After data analyses, radar charts were used to display the results.

### In vitro absorption, distribution, metabolism and excretion (ADME) evaluation

The ADME properties of QL-1200186 in vitro were measured by the following assays: Caco-2 permeability assay, CYP inhibition assay, protein binding assay, and liver microsome assay. Each assay was performed as previously described [25].

### Reporter gene assay

HEK-Blue™ IFN-α/β cells were used to determine the activity of QL-1200186. After 24 h of incubation with QL-1200186, HEK-Blue™ IFN-α/β cells (Invivogen; NKB-IFNAB) were stimulated with human IFNα (200 U/mL; catalog number: 11200–2; PBL Assay Science; Piscataway, NJ, USA) for 6 h. Stimulation of HEK-Blue IFN-α/β cells with human IFNα activates the JAK/STAT/ISGF3 pathway and subsequently induces the production of SEAP. Levels of SEAP in the supernatant were readily assessable using QUANTI-Blue™ Solution (InvivoGen, San Diego, CA, USA).

### Phosphorylation of STAT5 in human cells

With regard to IFNα-stimulated phosphorylation of STAT5 in human peripheral blood mononuclear cells (PBMCs), after incubation with QL-1200186 for 1 h, human PBMCs were stimulated with recombinant human IFNα (1000 U/mL; 11200–2; PBL Assay Science) for 30 min.

With respect to GM-CSF-stimulated JAK2 activation in U937 cells (Cobioer Biosciences, CBP60277), after 1 h of treatment with QL-1200186, U937 cells were stimulated with GM-CSF (20 ng/mL; C003; Novoprotein, Suzhou, China) for 15 min.

With regard to IL-2-stimulated JAK1/3 activation in human PBMCs, after 1 h of incubation with QL-1200186, human PBMCs were stimulated with human IL-2 (20 ng/mL; C013; Novoprotein) for 20 min.

With respect to IL-2-stimulated JAK1/3 activation in human whole blood cells, whole blood (50 μL) was preincubated with compounds for 30 min and then stimulated with IL-2 (20 ng/mL) for 15 min.

The stimulations stated above were terminated with prewarmed Lyse/Fix Buffer (558,049; BD Biosciences, San Jose, CA, USA) for 10 min. Cells were stained with an anti-cluster of differentiation (CD)3 fluorescein isothiocyanate (FITC) antibody (required for PBMCs but not for the other cell types), washed, and permeabilized on ice using Perm III Buffer (558,050; BD Biosciences) prior to staining with an Alexa Fluor 647 anti-phosphorylated (p)STAT5 (pY694) antibody for 30 min and analyzed by flow cytometry. Phosphorylation of STAT5 was quantified by the median fluorescence intensity (MFI) after gating on the CD3-positive population.

### IL-23 induced STAT5 phosphorylation in human Th17 cells

Human naïve CD4^+^ T cells were separated using a human naïve CD4^+^ T-cell isolation kit (StemCell, #17,852), and then cells were stimulated with anti-CD3 monoclonal antibody and anti-CD28 monoclonal antibody for 7–10 days under the following Th17-skewing treatments: IL-6, IL-23, IL-1β and transforming growth factor-β. Half of the medium was replaced with fresh medium containing a stimulant to induce differentiation every other day. Th17 cells were incubated overnight at 37 °C for deactivation. Cells were reinoculated on 96-well plates and cocultured with gradient-diluted QL-1200186 for 1 h and then stimulated with recombinant human IL-23 (ACRO: #IL-B-H52 WS) for 0.5 h. Cells were collected, and phosphorylation of STAT3 was quantified according to the MFI.

### IL-12/IL-18-induced IFNγ production in NK-92 cells and human whole blood cells

The inhibitory effect of QL-1200186 on IL-12/IL-18-induced IFNγ production in NK cells or human whole blood was determined by enzyme-linked immunosorbent assay (ELISA). NK92 cells were stimulated with recombinant human IL-12 (2 ng/mL; 200–12; PeproTech, Cranbury, NJ, USA) and recombinant human IL-18 (5 ng/mL; 9124-IL-050/CF; R&D Systems, Minneapolis, MN, USA) with or without QL-1200186 for 24 h at 37 °C. For human whole blood cells, whole blood (200 μL) was preincubated with the compound for 1 h and then stimulated with recombinant human IL-12 (2 ng/mL) or recombinant human IL-18 (10 ng/mL) for 24 h. After centrifugation, the supernatant was collected, and IFNγ production in the supernatant was measured with human IFNγ ELISA kits according to the manufacturer’s instructions (Biolegend, 430,104).

### TYK2 activation in Jurkat cells

Cells were cocultured with QL-1200186, BMS-986165 or NDI-034858 for 1 h and stimulated with recombinant human IFNα (1000 U/mL) for 15 min. Then, the phosphorylation of Tyr-1054 and Tyr-1055 in Jurkat cells (Cobioer Biosciences: CBP61444) was detected by western blotting to determine the inhibitory effect of QL-1200186 on TYK2 receptor-mediated activation.

### Phosphorylation of STAT1 in human PBMCs

Human PBMCs were preincubated with QL-1200186, BMS-986165 or NDI-034858 for 30 min and then stimulated with recombinant human IFNα (1000 U/mL) for 15 min. The reaction was terminated by collecting cells for surface staining. Cells were stained with anti-CD3 FITC (300406; Biolegend, San Diego, CA, USA), anti-CD19 APC (555415; BD Biosciences) or aqua fluorescent reactive dye BV421 (L34966A; Invitrogen, Carlsbad, CA, USA) antibodies for 20 min. After washing, fixation buffer (557870; BD Biosciences) was added and incubated for 10 min. Cells were washed and permeabilized on ice using Perm III Buffer (558050; BD Biosciences). Cells were stained with PE anti-PSTAT1 antibody (562069; BD Biosciences) for 30 min and analyzed by flow cytometry.

## IFNα-induced differentiation of human monocytes to dendritic cells

First, monocytes in human PBMCs were isolated using an EasySep™ Human CD14 Positive Selection Kit II (17858; Stemcell Technologies, Vancouver, Canada). Then, cells were seeded at 300,000 in 96-well plates and stimulated with GM-CSF (100 ng/mL; C003; Novoprotein), recombinant human IL-4 (50 ng/mL; 6507-IL-010/CF; R&D Technologies) plus IFNα (1000 U/mL; 11200–2; PBL Assay Science) for 6 days with or without QL-1200186, BMS-986165 or NDI-034858 at 5 nM, 20 nM or 100 nM. The media were half-changed on days 3 and 5. On day 6, cells were collected and stained with BV421 aqua fluorescent reactive dye (L34966A; Invitrogen), FITC mouse anti-human CD80 (555683; BD Biosciences), PE mouse anti-human CD86 (560957; BD Biosciences) or BUV737 mouse anti-human CD83 (612823; BD Biosciences) antibodies for 20 min. After staining, cells were washed twice and analyzed by flow cytometry.

## Thrombopoietin (TPO)-stimulated phosphorylation of STAT3 and STAT5 in platelets

After 30 min of preincubation of QL-1200186, BMS-986165, NDI-034858 or tofacitinib with whole blood cells, stimulation was undertaken using recombinant human TPO (50 ng/mL; 300–18; PeproTech) for 15 min. Stimulation was terminated by adding Lyse/Fix buffer. Staining with BUV786 mouse anti-human CD61 antibody (744384; BD Biosciences) was carried out, followed by washing, permeabilization and staining with PE mouse anti-human STAT3 antibody (651004; BD Biosciences) and APC mouse anti-human STAT5 antibody (562076; BD Biosciences), as described above. The expression of p-STAT3 and STAT5 was quantified by the MFI after the number of CD61-positive platelets was optimized.

### Pharmacokinetic (PK) study

The PK study was conducted using healthy male C57BL/6 mice (18–22 g, *n* = 6). Mice were fed and given free access to water prior to dosing. QL-1200186 was administered via oral or intravenous routes at doses of 10 mg/kg and 1 mg/kg, respectively. Blood samples were collected at 0.0833, 0.25, 0.5, 1, 2, 4, 8 and 24 h from the saphenous vein. Plasma was separated within 1 h of sampling by centrifugation at 1000 g. Liquid chromatography–tandem mass spectrometry of the sample was carried out on a mass spectrometer (API 5500; AB Sciex, Framingham, MA, USA). Chromatographic separation was achieved using a C18 column (2.6 µm, 50 × 3.0 mm; Kinetex; Phenomenex, Torrance, CA, USA). The main PK parameters were calculated by WinNonlin 8.0 (Certara, Princeton, NJ, USA).

### IL-12/IL-18-induced IFNγ release in mice

Healthy female, specific pathogen-free C57BL/6 mice (6–8 weeks) were intraperitoneally (i.p.) injected with IL-12 and IL-18 to drive IFNγ production. Mice were numbered, weighed and divided randomly into seven groups of five according to body weight using Excel™ (Office™; Microsoft, Redmond, WA, USA). One hour after drug administration, IL-12 (0.01 μg/mouse) was i.p. injected. One hour later, recombinant IL-18 (0.1 μg/mouse) was injected. Whole blood was collected 3 h later and centrifuged (300 g, 10 min, 4 °C), and serum was collected. IFNγ expression in serum was determined by a CBA Flex kit (BD Biosciences).

### Imiquimod (IMQ)-induced mouse model of psoriasis

Healthy male BALB/c mice (18–20 g) were divided randomly into three groups of eight according to body weight using Excel™ (Office™; Microsoft). After 1 week of adaptive feeding, mice received a daily topical dose of 62.5 mg of 5% imiquimod cream (20 mg; Aldara®; 3 M Pharmaceuticals, Maplewood, MN, USA) on the shaved back and left ear for 7 consecutive days. Control mice were treated similarly with vehicle cream (Vaseline lanette creme; Fagron, Amsterdam, the Netherlands). Mice in the treatment group were given QL-1200186 (5 mg/kg or 1 mg/kg) orally twice a day (p.o., b.i.d.).

The Psoriasis Area and Severity Index (PASI) consists of erythema, scaling and skin thickness measurements. Mice were evaluated from day 1 when imiquimod was given for 7 consecutive days. The thickness of the mouse skin was measured using a micrometer. The PK parameters were also measured.

### Statistical analyses

Data were analyzed using Prism 8.0.2 (GraphPad). One-way ANOVA was used to measure significant differences between multiple groups if a single factor was used. Two-way ANOVA with Bonferroni posttests was used if two factors were used. *P* < 0.05 was considered significant.

## Results

### QL-1200186 strongly binds to TYK2 JH2 with high selectivity

QL-1200186 (5^6^-methoxy-N-methyl-5^5^-(1-methyl-1H-pyrazol-3-yl)-8-oxa-2,4-diaza-1(2,6),3(2,4)-dipyridina-5(1,3)-benzenacyclononaphane-3^5^-carboxamide, which has a Simplified Molecular Input Line Entry System (SMILES) notation of O = C(NC)C1 = CN = C(N2)C = C1NC3 = CC(CCOCC4 = NC2 = CC = C4) = CC(C5 = NN(C)C = C5) = C3OC), was discovered during a project initiated by Shanghai Qilu Pharmaceutical R&D Center Limited (Shanghai, China). The project was outsourced to our contract research partner, Bioduro-Sundia (San Diego, CA, USA), where all medicinal, computational and chemistry work took place (patent number: WO 2022/213980 A1). Data on the structure–activity relationship of compound QL-1200186 will be disclosed in due course.

The binding mode of QL-1200186 is shown in Fig. 1B. QL-1200186 interacts with the TYK2 JH2 domain mainly through two hydrogen-bond networks. One occurs at the hinge region and involves the backbone carbonyl and NH groups of Val690. The other includes the backbone carbonyl group of Glu688 and the side chain of Lys642. QL-1200186 also showed additional aromatic hydrogen bonds to the backbone carbonyl groups of Glu688 and Glu691, which reinforces the interaction network.

First, we verified the biochemical binding of QL-1200186 to TYK2 in vitro. QL-1200186 could bind to the recombinant TYK2 JH2 domain with a half-maximal inhibitory concentration (IC_50_) of 0.06 nM and to the JAK1 JH2 domain at an IC_50_ of 9.85 nM. The selectivity of QL-120086 for the TYK2 JH2 domain was 164-fold greater than that for JAK1 JH2 (Fig. 1C). QL-1200186 did not show inhibitory activity against TYK2 JAK1/2/3 JH1 kinases (Fig. 1D and Table 1). Furthermore, TYK2 was also highly selective across the human kinome, with inhibition rates below 50% for all 207 kinases tested (Fig. 1E). The in vitro off-target screening assay further confirmed the above results (Fig. 1F).

**Table 1:**
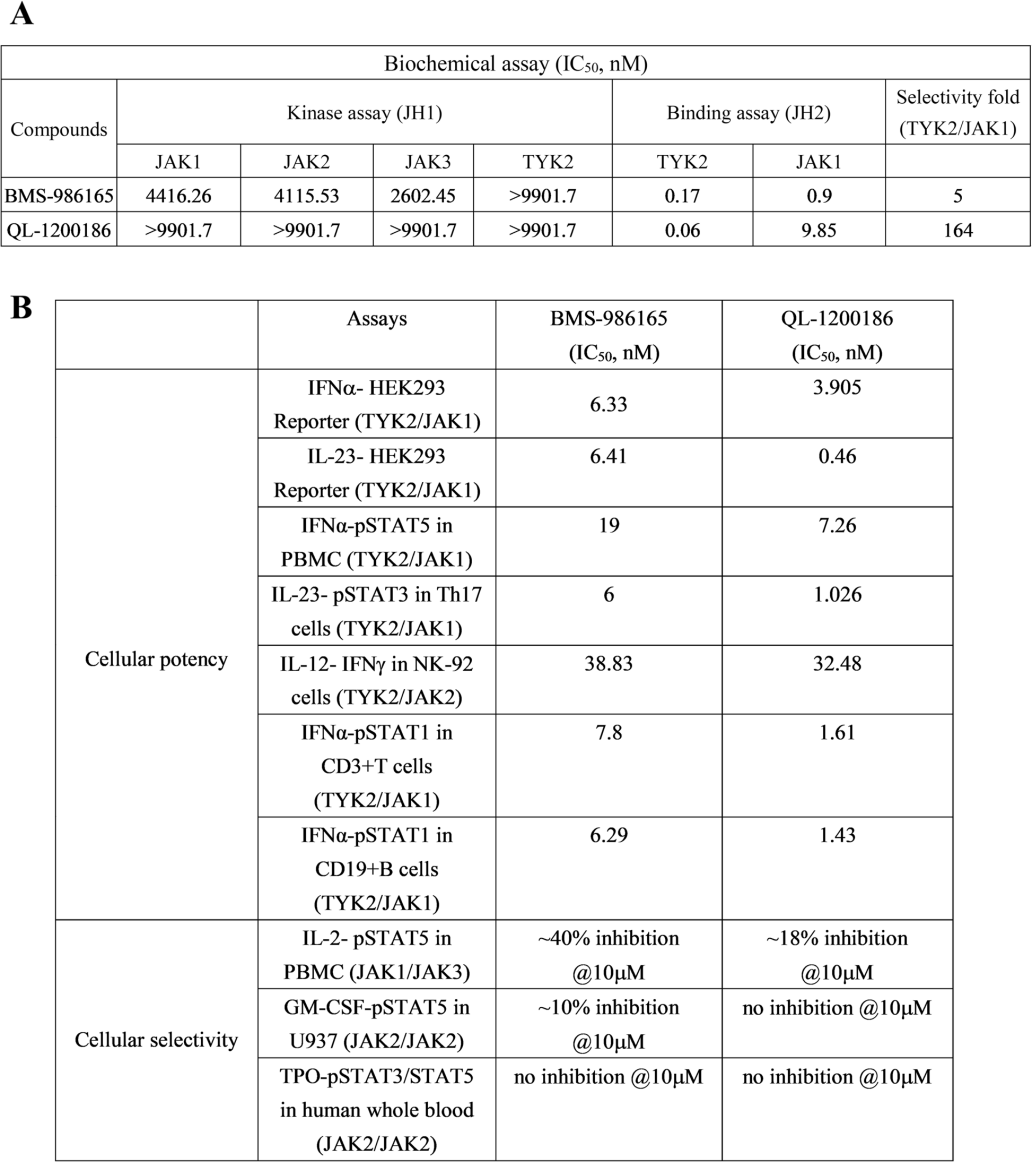
Biochemical binding and cellular potency of TYK2 inhibitors

The selectivity of QL-1200186 was also verified by functional phosphorylation assays. In the JAK2/JAK2-dependent GM-CSF-stimulated STAT5 phosphorylation assay, QL-1200186 demonstrated good selectivity with IC50 values of > 10 µM. A similar result was observed in the JAK1/JAK3-dependent IL-2-stimulated phosphorylation assay (Fig. 1G&H).

### QL-1200186 blocks the TYK2 signaling pathway

TYK2 can mediate signal transduction through the type-I IFN receptor, IL-12 receptor and IL-23 receptor. Hence, to further verify the function of QL-1200186 in the TYK2-mediated signaling pathway, we first examined the potency of QL-1200186 on human HEK-Blue IFN-α/β reporter cells and TYK2-mediated phosphorylation of STAT proteins in PBMCs.

**Fig. 2:**
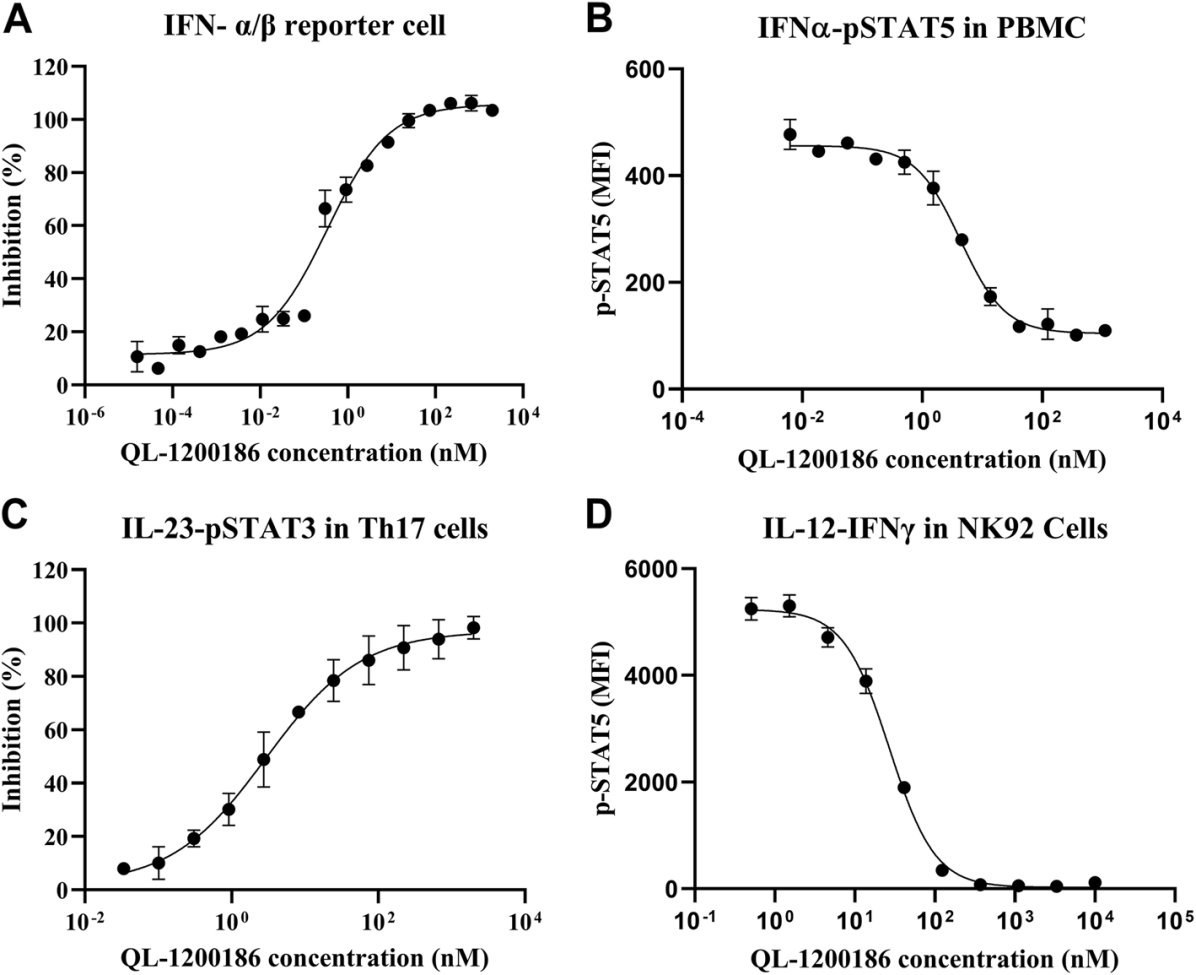
QL-1200186 blocks the TYK2 signaling pathway. **A** Effect of QL-1200186 on the JAK/STAT/ISGF3 pathway. **B** Human PBMCs were stimulated with human IFNα and pSTAT5 levels were determined. **C** Th17 cells were stimulated with IL-23 and pSTAT3 levels were determined. **D** IL-12 induced IFNγ production in NK92 cells. Data are the mean ± SD. At least three independent experiments were carried out

QL-1200186 showed dose-dependent inhibition of the JAK/STAT/ISGF3 pathway (Fig. 2A) and IFNα-stimulated pSTAT5 levels in CD3^+^ T cells (Fig. 2B). Considering the role of IL-23 in the TYK2-STAT signaling pathway, we further examined the effect of QL-1200186 on IL-23-induced phosphorylation of STAT3 (pSTAT3) in human Th17 cells. QL-1200186 inhibited the IL-23-induced pSTAT3 level in human Th17 cells in a dose-dependent manner (Fig. 2C), demonstrating that QL-1200816 had high potency on TYK2-STAT3 signaling mediated by the IL-23 receptor. Similarly, QL-1200186 had higher potency in IL-12-induced IFNγ production in NK92 cells (Fig. 2D). These data indicated that QL-1200186 mediated TYK2 inhibition in a highly selective fashion compared with JAK1/2/3 signaling pathway inhibition (Fig. 1G&H). IC_50_ values are shown in Table 1.

### Inhibitory function and selectivity of QL-1200186 compared with other TYK2 and JAK1/3 inhibitors

Deucravacitinib (BMS-986165) is a novel drug administered via the oral route. It is on the market and has been reported to block key molecules in the pathogenesis of psoriasis, including TYK2 [23]. NDI-034858 is an allosteric inhibitor of TYK2 developed by Nimbus. It is in phase II clinical trials for the treatment of plaque psoriasis and psoriatic arthritis [26]. Thus, to further explore the inhibitory function of QL-1200186, we compared the function and selectivity of QL-1200186 to those of BMS-986165 and NDI-034858.

**Fig. 3:**
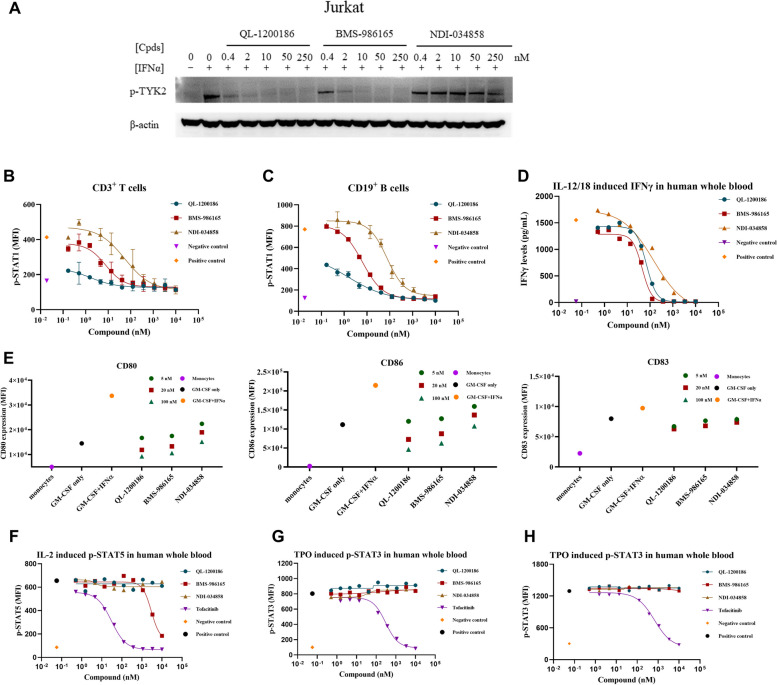
Inhibitory function and selectivity of QL-1200186 compared with other TYK2 inhibitors. **A** Effect of TYK2 inhibitors on IFNα-induced pTYK2 expression. **B**, **C** Effects of TYK2 inhibitors on pSTAT1 expression in CD3 + T and CD19 + B cells (*n* = 3). **D** Effects of TYK2 inhibitors on IL-12-induced IFNγ production in human whole blood cells (*n* = 3). **E** Effects of TYK2 inhibitors on the differentiation of monocytes into dendritic cells (*n* = 2). **F** Effect of a TYK2 inhibitor and tofacitinib on the activity of JAK 1/3 was detected by IL-2-induced pSTAT5 expression (*n* = 3). **G**, **H** Effect of compounds on JAK 2/2 activity was determined by thrombopoietin-induced pSTAT3/5 expression (*n* = 3)

First, we attempted to understand the direct effects of the three TYK2 inhibitors on TYK2 activation (Fig. 3A). QL-1200186 blocked IFNα-stimulated phosphorylation of TYK2 in Jurkat cells in a concentration-dependent manner. The blockade elicited by QL-1200186 was significantly greater than that elicited by NDI-034858 and comparable to that elicited by BMS-986165. Consistent with receptor-mediated TYK2 activation, QL-1200186 significantly inhibited IFNα-induced STAT1 phosphorylation in CD3^+^ T cells and CD19^+^ B cells in human PBMCs. QL-1200186 showed a superior inhibitory effect compared with that of the other two TYK2 inhibitors (Fig. 3B, C). Type-I IFN plays an important role in inducing monocytes to differentiate into antigen-presenting cells and is closely related to autoimmune diseases [27]. Hence, we further investigated the role of QL-1200186 and two other TYK2 inhibitors in the differentiation of monocytes into mature dendritic cells induced by GM-CSF and IFNα. According to the expression of CD80, CD83 and CD86 (Fig. 3E), QL-1200186 significantly inhibited the differentiation and maturation of monocytes into dendritic cells, and the inhibitory effect was greater than that of NDI-034858 or BMS-986165. In addition, in accordance with the previous result (Fig. 2D), IFNγ production was significantly reduced in human whole blood after treatment with QL-1200186, and the IC_50_ value was comparable to that of BMS-986165.

In addition, we compared the selectivity of QL-1200186, BMS-986165 and NDI-034858 for TYK2 and JAK1/2/3 in human whole blood assays. QL-1200186 and NDI-034858 showed no activity in IL-2-mediated JAK1/JAK3 signaling or thrombopoietin (TPO)-induced JAK2/JAK2 signaling (Fig. 3F–H). BMS-986165 showed a slight inhibitory effect on IL-2-induced JAK1/3 expression at high concentrations compared with that of tofacitinib, an approved JAK1/3 inhibitor.

Overall, compared with other TYK2 inhibitors (BMS-986165 and NDI-034858) and JAK1/3 inhibitors (tofacitinib), we found that QL-1200186 had excellent inhibitory function and selectivity against TYK2.

### QL-1200186 has a decent PK profile

Next, an in vitro ADME study of QL-1200186 was performed. As shown in Table 2, QL-1200186 had a suitable in vitro absorption and metabolic stability profile, with no specific human metabolites in vitro and no significant inhibition of primary cyp isoenzymes according to Met ID. Furthermore, in the mouse pharmacokinetic study, oral administration of QL-1200186 (10 mg/kg) could reach 20,320 h*ng/mL of the area under the curve, and the bioavailability was 137%, whereas intravenous administration (1 mg/kg) showed a lower clearance rate (11.6 mL/min/kg) and a low distribution volume of 0.842 L/kg (Table 3). Hence, QL-1200186 exhibited suitable PK properties, with excellent exposure and high bioavailability in vivo, which provides promising preclinical evidence for its further clinical development.

**Table 2:**
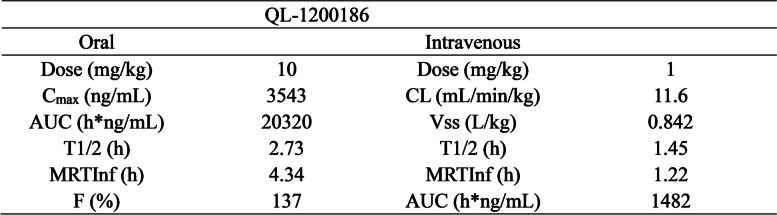
In vitro ADME parameters of QLS-1200186

**Table 3:**
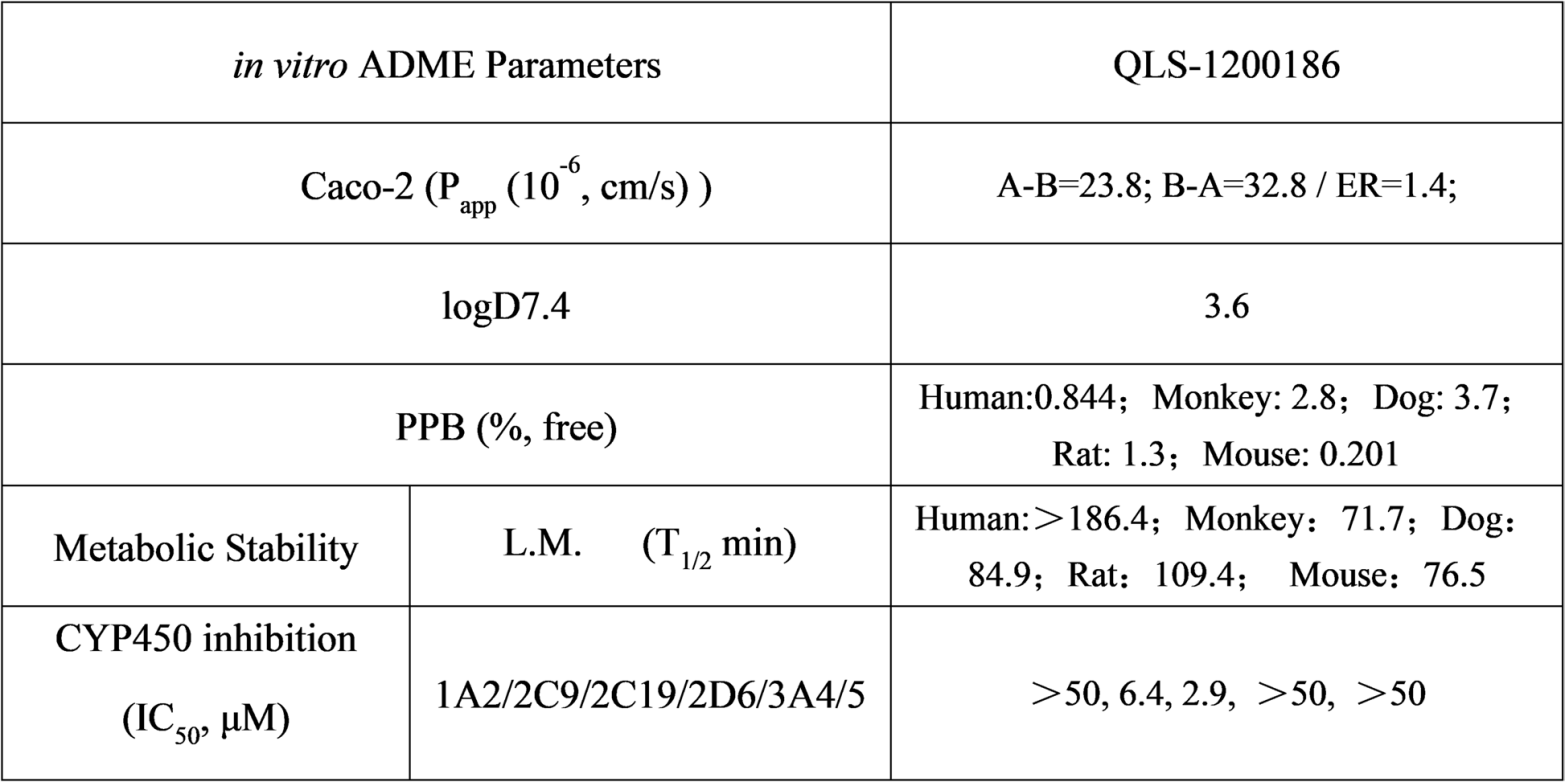
PK profiles for QL-1200186 in mice

Cmax: maximum concentration; AUC: area under the curve*;* MRTInf: mean residence time

F (%): The percentage (or the fraction F) of an administered dose of a xenobiotic that reaches the systemic circulation

### QL-1200186 reduces IL-12/IL-18-driven IFNγ release in vivo

**Fig. 4:**
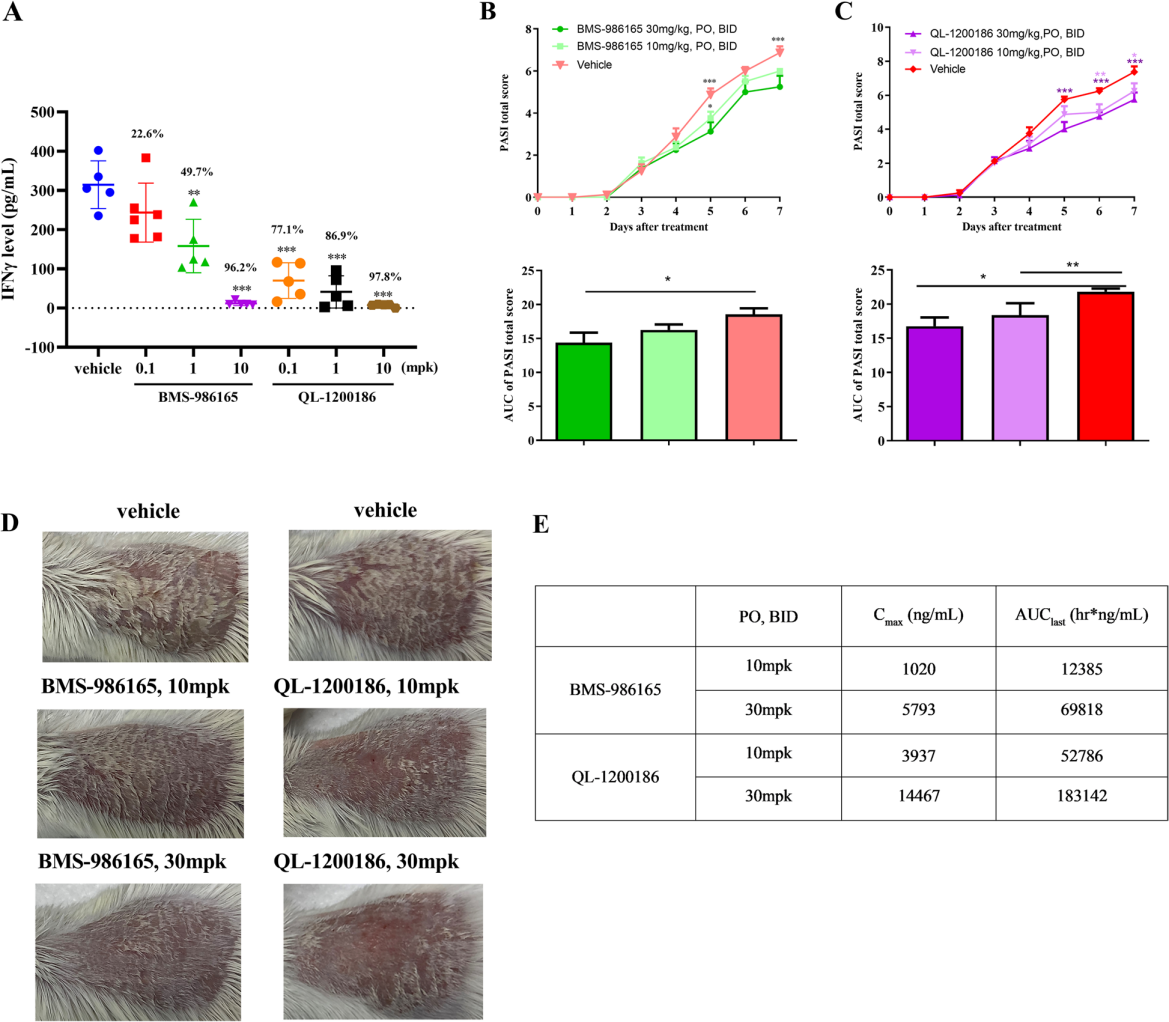
Effect of QL-1200186 against IL-12-driven responses and imiquimod (IMQ)-induced psoriasis-like skin inflammation in vivo. **A** Effects of QL-1200186 on serum IFNγ production in a mouse model induced by IL-12 and IL-18. **B**, **C** Total PASI scores and area under the curve (AUC) on day-7 in an imiquimod-induced psoriasis-like model (*n* = 8). **D** Representative images of skin from imiquimod-induced psoriasis-like model mice taken on day-7. **D** PK profiles of QL-1200186 in psoriasis-like model mice

The above results revealed that QL-1200186 potently and selectively blocked TYK2-related functional pathways and showed encouraging PK characteristics. Therefore, the compound was further functionally validated in model mice that had elevated TYK2-mediated IFNγ production. In this model, mice were i.p. injected with ILs associated with a TYK2-dependent receptor pathway (IL-12 and IL-18) to drive IFNγ production. Oral administration of QL-1200186 dose-dependently inhibited IFNγ production by 77.1%, 86.9% and 97.8% at doses of 0.1, 1 and 10 mg/kg, respectively (Fig. 4A), which was consistent with the in vitro results (Figs. 2D and 3G). BMS-986165 had an inhibitory effect on IFNγ production, but much less so than QL-1200186, indicating that QL-1200186 was a powerful inhibitor of TYK2 signaling.

### QL-1200186 can improve imiquimod-induced psoriasis-like skin inflammation in mice

To evaluate whether QL-1200186 could improve psoriasis, we treated model mice with psoriasis-like skin inflammation induced by imiquimod with QL-1200186. The PASI scores of mice in each group were evaluated every day, and the differences among groups were significant (*n* = 8). Compared with the imiquimod group, the QL-1200186 treatment group exhibited significantly improved psoriasis-like inflammatory symptoms such as erythema, scaling and thickening (Fig. 4B–D). QL-1200186 significantly alleviated psoriasis severity according to the thickness score on day 7 (Fig. 4E). In general, QL-1200186 exerted excellent efficacy in improving psoriatic dermatosis.

## Discussion

In this study, we investigated a novel TYK2 allosteric inhibitor named QL-1200186, which was developed by Qilu Pharmaceuticals. First, the molecular docking results showed that QL1200186 binds to TYK2 JH2 and JAK1 JH2 at two different residues in the binding sites: ILE597 (VAL603 in TYK2) and GLU667 (THR687 in JAK1). The different binding residues results in the high selectivity of QL1200186 to TYK2 JH2. Additionally, we demonstrated that QL-1200186 had a robust inhibitory effect on TYK2-mediated proinflammatory signaling, including IFNα-induced phosphorylation of STAT5, IL-12-stimulated IFNγ secretion and IL-23-mediated phosphorylation of STAT3 in immune cells. Conversely, QL-1200186 exhibited no or little inhibition of JAK2/JAK2-dependent GM-CSF-stimulated or JAK1/JAK3-dependent IL-2-stimulated phosphorylation of STAT5 in PBMCs. Moreover, QL-1200186 significantly inhibited IL-12/IL-18-induced IFNγ production in vivo and alleviated IMQ-induced psoriasis-like skin inflammation in mice, which confirms the importance of these pathways in psoriasis [19, 28].

The JAK family contains JAK1, JAK2, JAK3 and TYK2. Accumulating evidence shows that JAK1, JAK2 and JAK3 regulate multiple pathways, including cytokine signaling, growth hormone signaling, and erythropoietin signaling. Several JAK inhibitors targeting the JH1 domain have been approved to treat inflammatory diseases, including psoriasis. However, the use of these pan-JAK inhibitors is limited due to clinical adverse effects, including an increased risk of infection, lymphocytopenia, thromboembolism, dyslipidemia, altered liver function metabolism, and even an increased risk of cancer [29–33]. These adverse effects are associated with the inhibition of JAK1-JAK3. Therefore, developing molecules with high selectivity for TYK2 provides a unique approach with potential advantages.

In recent years, inhibition of TYK2 by targeting the JH2 domain has been reported. Deucravacitinib is the first drug of this new class of inhibitors that was approved by the FDA [34]. Although deucravacitinib showed greater selective inhibition of TYK2 than JAK1/JAK2/JAK3, binding to the JH2 domain of JAK1 and other subtypes of the JH1 domain (JAKs) to varying degrees has been reported [19, 28, 35]. Conversely, QL-1200186 binds very weakly to the JH2 domain of JAK1 at different concentrations and has no effect on the JH1 domain of other homologous isomers, which means that QL-1200186 has a higher selectivity. Another allosteric TYK2 inhibitor, NDI-034858, which was acquired by Takeda, is in phase II clinical trials and has shown superior functionality and an encouraging activity profile. In this study, we demonstrated that QL-1200186 had greater cellular potency. Using a mouse model, we measured IFNγ production after treatment with murine IL-12 and IL-18. QL-1200186 significantly suppressed IFNγ production in a dose-dependent manner. Moreover, QL-1200186 showed a greater inhibitory effect at 0.1 mg/kg and 1 mg/kg doses than BMS-986165. In addition, QLS-1200186 reduced disease severity in an IMQ-induced psoriasis model. Future experiments that demonstrate the efficacy QL-1200186 in other immune-mediated inflammatory disease models will further support its clinical development.

Although there is compelling evidence that QL-1200186 can alleviate psoriasis, the urgent need for the development of safe drugs against autoimmune diseases remains unresolved. Off-target drug reactions are the main cause of adverse drug reactions. A considerable proportion of research and development projects testing new drugs stall and terminate at a late stage due to failing to detect off-target effects early in development, which causes clinical trials to fail due to adverse drug reactions. To accurately design drugs, improve the success rate of research and development projects and reduce the failure rate of clinical trials, we evaluated the in vitro off-target effects of QL-1200186 based on 44 early drug safety targets jointly proposed by AstraZeneca, GlaxoSmithKline, Novartis and Pfizer to determine off-target effects. QL-1200186 showed suitable safety at a dose of 10 µM. In addition, QL-1200186 showed excellent safety in toxicological studies on dogs and rats (data not shown).

## Conclusions

There is an unmet need for safe, rapid-acting and efficacious drugs against psoriasis and other autoimmune diseases. There is a strong rationale for targeting the TYK2 JH2 domain in these diseases, and QL-1200186 may be a promising option. QL-1200186 potently blocks the cytokine-mediated inflammatory response through TYK2. These preclinical pharmacological and safety assessments support its further clinical development and continued evaluation of its efficacy against autoimmune diseases.

### Supplementary Information

**Additional file 1: Supplementary Figure S1. **QL-1200186 blocked IFNα-stimulated phosphorylation of TYK2 in Jurkat cells in a concentration-dependent manner. Cells were co-cultured with QL-1200186, BMS-986165 or NDI-034858 for 1 h and stimulated with recombinant human IFNα (1000 U/mL) for 15 min. The phosphorylation level of TYK2 in Jurkat cells was detected by western blotting. The PVDF membrane was clipped at 65kda. The upper membrane incubated P-TYK2 (A) and the lower membrane incubated β-actin. (C) and (D) are the corresponding maker photos of PTYK2 and β-actin, respectively.

## Data Availability

Data may be provided from the corresponding author upon reasonable request.

## Abbreviations

ADME: Absorption distribution metabolism excretion
AUC: Area under the curve
BID: Twice daily
CYP: Cytochrome P450
ELISA: Enzyme-linked immunosorbent assay
ER: Efflux ratio
GM-CSF: Granulocyte–macrophage colony stimulating factor
IBD: Inflammatory bowel disease
IFN: Interferon
IL: Interleukin
JAK: Janus kinase
MFI: Median fluorescence intensity
PASI: Psoriasis area and severity index
PBMC: Peripheral blood mononuclear cell
PK: Pharmacokinetic
PPB: Plasma protein binding
SEAP: Secreted alkaline phosphatase
SLE: Systemic lupus erythematosus
STAT: Signal transducer and activator of transcription
TYK2: Tyrosine kinase 2
