# Supplementary material for: A novel highly selective allosteric inhibitor of tyrosine kinase 2 (TYK2) can block inflammation- and autoimmune-related pathways
Source: Cell Commun Signal. 2023 Oct 16;21:287. doi: 10.1186/s12964-023-01299-7 (PMC10578023; doi:10.1186/s12964-023-01299-7)
Supplement: Supplementary file 2 — Additional file 1: Supplementary Figure S1. QL-1200186 blocked IFNα-stimulated phosphorylation of TYK2 in Jurkat cells in a concentration-dependent manner. Cells were co-cultured with QL-1200186, BMS-986165 or NDI-034858 for 1 h and stimulated with recombinant human IFNα (1000 U/mL) for 15 min. The phosphorylation level of TYK2 in Jurkat cells was detected by western blotting. The PVDF membrane was clipped at 65kda. The upper membrane incubated P-TYK2 (A) and the lower membrane incubated β-actin. (C) and (D) are the corresponding maker photos of PTYK2 and β-actin, respectively. [file 12964_2023_1299_MOESM1_ESM.pptx]

## Slide 1
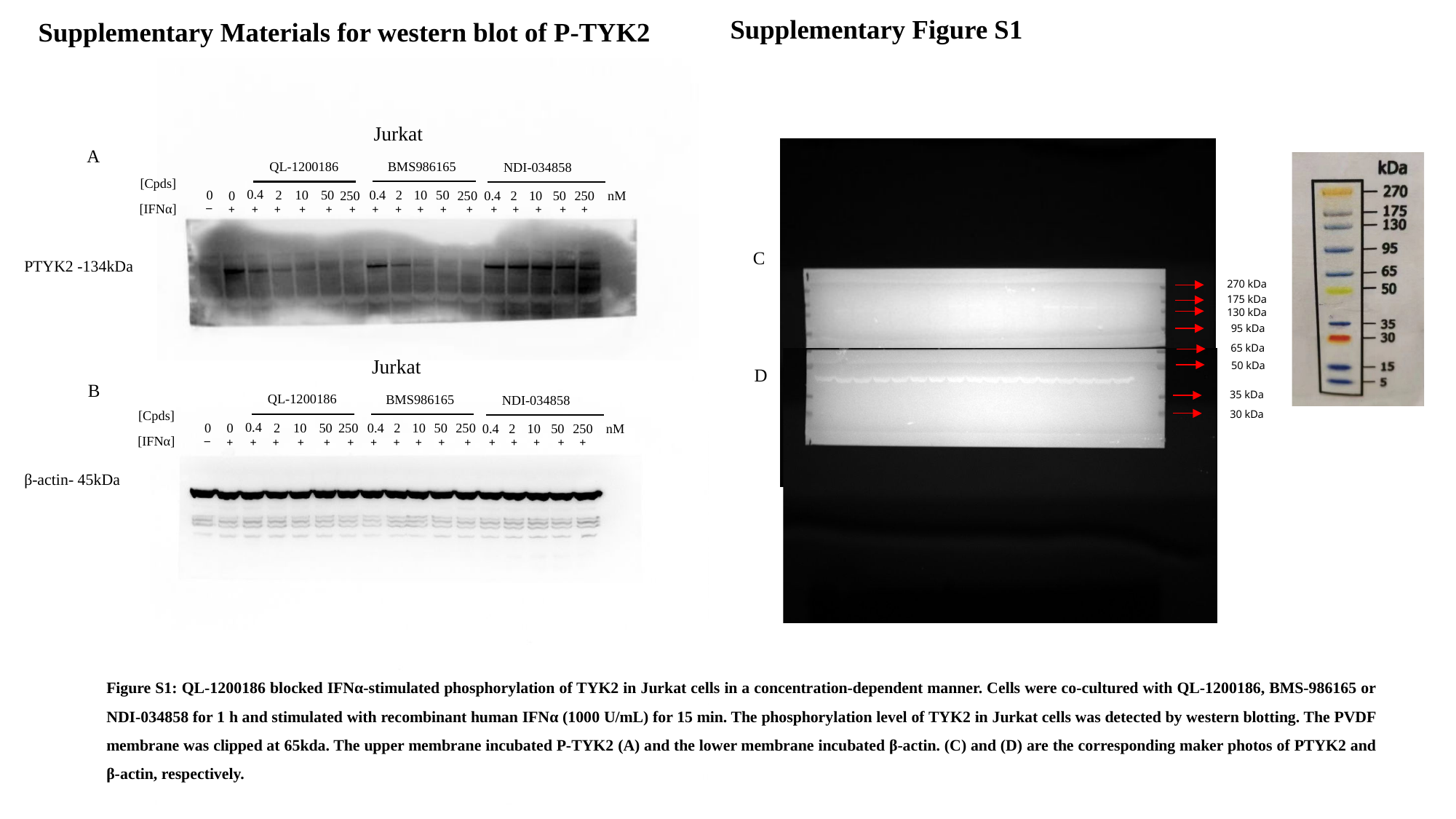

Supplementary Figure S1
Supplementary Materials for western blot of P-TYK2
Jurkat
250 nM
0.4
2
10
50
QL-1200186
0.4
2
10
50
250
BMS986165
0.4
2
10
50
250
NDI-034858
[Cpds]
0
0
_
[IFNα]
+
+
+
+
+
+
+
+
+
+
+
+
+
+
+
+
PTYK2 -134kDa
270 kDa
175 kDa
130 kDa
95 kDa
65 kDa
Jurkat
250 nM
0.4
2
10
50
QL-1200186
0.4
2
10
50
250
BMS986165
0.4
2
10
50
250
NDI-034858
[Cpds]
0
0
_
[IFNα]
+
+
+
+
+
+
+
+
+
+
+
+
+
+
+
+
 50 kDa
35 kDa
30 kDa
β-actin- 45kDa
A
C
D
B
Figure S1: QL-1200186 blocked IFNα-stimulated phosphorylation of TYK2 in Jurkat cells in a concentration-dependent manner. Cells were co-cultured with QL-1200186, BMS-986165 or NDI-034858 for 1 h and stimulated with recombinant human IFNα (1000 U/mL) for 15 min. The phosphorylation level of TYK2 in Jurkat cells was detected by western blotting. The PVDF membrane was clipped at 65kda. The upper membrane incubated P-TYK2 (A) and the lower membrane incubated β-actin. (C) and (D) are the corresponding maker photos of PTYK2 and β-actin, respectively.
